# Cognitive Predictors of Everyday Functioning in Older Virally Suppressed Women with HIV in the Women’s Interagency HIV Study

**DOI:** 10.21203/rs.3.rs-6136690/v1

**Published:** 2025-03-10

**Authors:** David E. Vance, Lang Lang, Pauline M. Maki, Danyang Yu, Raha Dastgheyb, Yuezhe Wang, Gayle Springer, Kathryn Anastos, Deborah R. Gustafson, Kathleen M. Weber, Derek M. Dykxhoorn, Joel Milam, Monica M. Diaz, Seble G. Kassaye, Drenna Waldrop, Yanxun Xu, Leah. H. Rubin

**Affiliations:** University of Alabama at Birmingham; Johns Hopkins University; University of Illinois at Chicago; Johns Hopkins University; Johns Hopkins University School of Medicine; Johns Hopkins University; Johns Hopkins Bloomberg School of Public Health; Albert Einstein College of Medicine and Montefiore Medical Center; State University of New York Downstate Health Sciences University; Cook County Health and Hektoen Institute of Medicine; Dr. John T. Macdonald Foundation, University of Miami; University of California; University of North Carolina at Chapel Hill; Georgetown University; Emory University; Johns Hopkins University; Johns Hopkins University School of Medicine

**Keywords:** HIV, cognition, motor function, instrumental activities of daily living, activities of daily living

## Abstract

In the Women’s Interagency HIV Study, we examined the association between objective cognitive test performance and the self-rated Lawton and Brody scale of Independent Activities of Daily Living (IADL) in 754 older (50+) women with HIV (WWH; 84% virally suppressed). To handle this longitudinal data, weighted logistic mixed effect models examined associations between cognitive domain performance (predictor) and functional outcomes (IADL item level scores). In the total sample, poorer motor performance was associated with impairments in *home repairs*, *housekeeping*, and *laundry* and poorer executive functioning was associated with impairment in *planning social activities*. Among older virally suppressed-WWH, poorer motor performance was associated with deficits in *home repair* and poorer executive performance was associated with deficits in *planning social activities*. Since motor and executive performance were related to impairments in certain IADLs, strategies such as cognitive training targeting these domains could improve everyday functioning.

## Introduction

From a meta-analysis of 18 studies, the prevalence of HIV-Associated Neurocognitive Disorder is 44.9% in people with HIV (PWH) [1]. Fortunately, more severe forms such as HIV-related dementia have decreased markedly with advancements in antiretroviral therapy (ART), yet milder forms of cognitive impairment remain [2]. One subgroup particularly at risk for cognitive impairment is women with HIV (WWH) [3], which comprise nearly 21% of the HIV population in the United States [4] and 53% of the HIV population worldwide [5]. The strongest evidence from adequately powered studies indicates that WWH show greater cognitive deficits globally but also in the domains of learning and memory followed by processing speed and motor function compared to their uninfected peers [6, 7]. As the population of PWH is expected to age and experience age-related cognitive declines, concerns grow that such existing cognitive impairments will become more severe and may be exacerbated in already cognitively vulnerable subgroups such as WWH [8]. These cognitive impairments can develop into everyday functional impairment in either basic activities of daily living (BADL; e.g., bathing, dressing), instrumental activities of daily living (IADL; e.g., grocery shopping, paying bills), or both [9, 10].

Impairments in activities of daily living remain a source of severe stress for both PWH and their support system (including family, friends, and formal/informal caregivers), and can result in serious physical, emotional, and financial burden. Subjective, self-rated functional abilities are often measured using the Lawton & Brody IADL scale while objective measures use timed or performance-based tasks. The prevalence and severity of IADL impairments increase with age and cognitive impairment. For example, Johs et al. examined the Lawton & Brody IADL scale in association with a frailty measure [11] among 1,015 PWH participating in the AIDS Clinical Trials Group A5322 HAILO study (median age 51 years, 15% older than 60). Six percent of respondents self-reported impairments in two or more IADLs and an additional 11% had one impaired IADL. Approximately 20% of participants over age 65 were classified as functionally impaired (versus 6–8% for NHANES data in a non-HIV population). The most common impairments were with housekeeping (48%), transportation (36%), and shopping (28%), while the least frequently endorsed were medication management (5%) and telephone use (12%). Lower scores on a cognitive screener (i.e., Neuroscreen) were significantly associated with IADL impairment, although the impact of impairment in different cognitive domains was not reported.

Different components of the IADL scale are likely to be associated with different underlying cognitive impairments, especially as people age. For example, in 26 middle-aged and older PWH, better processing speed was required for better driving simulator performance [12]. Similarly, in a different study of 145 younger and 119 older PWH, Fazeli et al. observed that better logical memory was related to better understanding reading or TV, but only in the older (50+) PWH group [13].

In this exploratory study, we examined neuropsychological test performance in older (50 + years) WWH from the Women’s Interagency HIV Study (WIHS) to test the relationship between objective cognitive functioning and the subjective Lawton & Brody IADL scale in an item-by-time analysis for each of the ADLs and IADLs. Functioning across seven cognitive domains were examined to assess their relationship to self-reported impairments in each of the Lawton & Brody everyday functioning items including housekeeping, managing finances, buying groceries, cooking, and so forth. Based on the cognitive demands of everyday functioning tasks, our study aim was to explore whether specific cognitive domains would be associated with specific components of the IADL scale. Furthermore, we also examined whether being virally suppressed would contribute to these relationships between cognitive domains and components of the IADL scale. Now that viral suppression is more commonplace in PWH, cognitive sequalae has been reduced but not eliminated. Even when virally suppressed, low-grade inflammation is still present which can impact multiple organ systems include the central nervous system and the brain [1]. Thus, it is important to examine the relationships between everyday functioning and cognition in those who are virally suppressed. This study addresses a crucial need for developing remediation programs that would allow PWH to compensate for their everyday functional impairments by understanding each activity as a unique entity requiring different cognitive efforts.

## Methods

The WIHS is a multi-center, prospective study examining the natural progression of HIV in WWH. The first wave of study enrollment occurred between October 1994 and November 1995, October 2001 and September 2002, and January 2011 and January 2013 at six sites (Brooklyn, Bronx, Chicago, DC, Los Angeles, and San Francisco). A more recent enrollment wave occurred when new sites were added specifically in high HIV prevalence areas of the southern US (Chapel Hill, Atlanta, Miami, Birmingham, and Jackson) between October 2013 and September 2015. Detailed information about study methodology of the WIHS including ethical approval, consenting, information regarding recruitment processes and eligibility criteria, tester training, and quality assurance procedures have been previously published [14–16]. The present longitudinal mixed-effects model analysis was restricted to WWH 50 and older who had completed a battery of neuropsychological tests and a modified Lawton & Brody IADL scale. The Lawton & Brody IADL scale was implemented in the WIHS in April 2013. There were 2,231 participants with 5,131 visits of IADL data. Out of the 2,231 participants, 2,025 (91%) had completed the neuropsychological test battery and had relevant covariate data available (total visits=4,382). Among the 2,025 participants, 1,062 were above 50 years old (total visits=2,079). Of the 1,062 participants above 50 years old, 754 were WWH with 1,457 visits and 637 of these WWH were virally suppressed with 1,080 visits. Participants were required to be biologically female to enter the study; there were no transgendered women in the study.

### Neuropsychological Test Battery and Outcomes

The battery of neuropsychological tests assessed seven domains [17]; *T*-scores for each of the measures in each domain were averaged. ***Verbal Learning*** and ***Memory*** were assessed with the Hopkins Verbal Learning Test-Revised (HVLT-R; outcomes=total learning, delayed free recall) [18]. ***Verbal Fluency*** was assessed with the Controlled Oral Word Associations Test (COWAT; outcome=total correct words generated across three trials [F, A, S]) and Animal fluency (outcome=total correct animals generated). ***Attention/Working Memory*** was assessed with the Letter-Number Sequencing (LNS; outcomes= total correct on the working memory and attention conditions). ***Psychomotor Speed*** was assessed with the Symbol Digit Modalities Test (SDMT (written); outcome=total correct) and the Stroop test (outcome=time to complete Trials 2 [color naming]). ***Executive Function*** was assessed with Trail Making Test (TMT; outcomes=time to complete Part B) and the Stroop test (outcome=time to complete 3 [color-word]). ***Motor Function*** was assessed with the Grooved Pegboard (GPEG; outcomes=time to completion, dominant and non-dominant hand).

Timed outcomes were log transformed to normalize distributions and reverse scored so higher scores equated to better performance. Demographically-adjusted *T*-scores were calculated for each outcome [19, 20]. *T*-scores were normalized to have an average of 50 and a standard deviation of 10. Mean *T*-scores between 45 and 55 are considered within the normal range. These *T*-scores were then used to create domain scores.

### Instrumental Activities of Daily Living (IADL)

A modified Lawton & Brody IADL scale was implemented in the Women’s Interagency HIV Study; although men also provide *childcare*, it was modified to include items specific to women (i.e., *childcare*) as they have been traditionally identified as engaging in this activity. This is a self-report assessment of functional status in adults covering several functional domains including *cooking*, *grocery shopping*, *housekeeping*, *handling money*, and more. Queries were asked about how well (i.e., ranging from “best ever”; 1 – 4 with 1 indicating highest level of independence to 4 indicating “unable to perform”) the participant has been able to perform in these functional domains as well as currently how able one is to perform in these functional domains now (in the last month; 1 – 4 with 1 indicating highest level of independence to 4 indicating “unable to perform”). To clarify, this information was asked when participants first entered the study and then at subsequent follow up visits with the explicit purpose to compare historically their overall performance in order to measure self-rated decline over time.

Identical to the Multicenter AIDS Cohort Study (MACS) [21], a global (total) classification of normal, mild, or pronounced impairment for IADLs was made if at least 14 of 16 IADL items were completed. Severe impairment was defined as a major deficit on two or more items or a combination of major/minor deficits on four or more items. Mild impairment was defined as a minor deficit on two or more items or a combination of major/minor deficits on two or more items (without qualifying for pronounced impairment). Individuals not meeting either criteria for major or minor impairment were classified as normal. For the present study, we combined mild and pronounced impairment because the percent of mild impairment was low; we did this so we could further explore the relationship between cognition and each of the items of the Lawton & Brody and increase our power to detect differences; this resulted in 38% for the entire sample and 35% for the virally suppressed WWH (Table 2).

In addition to the global classification of IADL impairment, we also determined item-level IADL impairment using the historical “best ever” score and current score for that item. Specific IADL deficits were first calculated from subtracting ratings in the last month from the “best ever” functioning rating. Higher negative scores indicated a decrease in function from “best ever” to current. Using these values, a specific IADL was considered impaired if: 1) the difference between the “best ever” score and current score was ≤ −2; or 2) the difference between the “best ever” score and current IADL was −1 and the current IADL was already the lowest value in the scale (e.g., 2 or 3) for that specific IADL.

### Antiretroviral Adherence

Adherence to ART was based on two self-reported items. The first item assessed frequency of ART intake as prescribed over the past 6 months, and the second item queried ART use at the study visit. A 5-level Likert scale was used to quantify responses to the first item and included: 1=“100% of the time”, 2=“95–99% of the time”, 3=“75–94% of the time”, 4=“<75% of the time”, or 5=“I haven’t taken any of my prescribed medications.” Adherence was defined as self-reported ART adherence at the study visit and at least 95% in the past six months; these were averaged over the study visits.

### Covariates

Several sociodemographic (e.g., race/ethnicity, age, years of education), behavioral, and clinical factors were examined as potential confounders of the associations between cognitive performance and IADL outcomes, as is common in many of our WIHS cognitive analyses [17]. Based on our prior analyses showing that they exert a relationship to cognitive performance, the following covariates were selected and statistically controlled in all models to derive a more precise evaluaton of the relationship between cognition IIV and IADL outcomes: clinic site, age, years of education, annual household income, depressive symptoms via the Center for Epidemiological Studies Depression scale (CES-D), body mass index, current smoker, recent heavy alcohol use, and recent illicit substance use (marijuana and crack use); race/ethnicity was not included as a covariate. Additional HIV-related covariates were included: nadir and current CD4 count, prescribed ART therapy, ART adherence and duration of use, and previous AIDS diagnosis.

### Statistical Analyses

A series of longitudinal weighted logistic mixed effect models (with random intercept) were conducted to examine associations between cognitive function (predictor) and IADL total and item level scores; the repeated visits from the same participants were averaged as the models accounted for the multiple visits. We omitted from our item analysis *bathing* and *using the phone* because there was little impairment (<1%) in our data. For each model, the seven cognitive domain scores were included as predictor variables as well as the identified confounders. Initial models were conducted in the: WWH and virally suppressed-WWH. All models were conducted using the package *lme4* in R version 4.1.2. Since this was an exploratory analysis, significance was set at *P*≤0.01.

## Results

### Sample Characteristics

There were 754 WWH (*n*=637; 84% virally suppressed) in this total sample (Table 1). In the total sample, 73% were non-Hispanic Black; mean age approximately 55.03 years; 12.49 years of education; and 54% with an annual household income of ≤$12,000. Overall, 40% were current smokers and 19% used marijuana. On average WWH were on ART for 12.9 years and 87% were ART adherent (≥95%). The most common ART used by WWH included Emtricitabine (69%), Tenofovir (51%), and Ritonavir (a boosting agent, 33%). Comparisons between virally-suppressed WWH to those without revealed no significant differences, except on adherence rates with virally-suppressed WWH having a higher prevalence of adherence (90% vs 87%).

With respect to IADLs, approximately 38% of the total sample showed an overall impairment in IADLs; however, as expected this impairment (35%) was slightly less in virally suppressed. With respect to cognitive function, individual domain *T*-scores approached 50. Although the differences were small, as expected virally-suppressed WWH demonstrated better performance overall across domains (Table 2) but this was only statistically significant for *laundry*. *Working*, *housekeeping*, *home repairs*, *social activities*, and *laundry* were among the most common IADLs to be more impaired in the overall sample.

### Cognitive Domains Predicting Everyday Functioning

**Figure 1:**
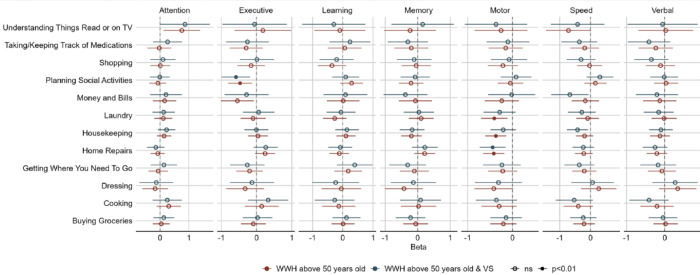
Associations between cognitive domains and instrumental activities of daily living (IADL) in older [>50 years of age] women with HIV (WWH) and virally-suppressed (VS) women with HIV (WWH). A negative beta coefficient from the models indicate that better cognition is associated with a decrease in IADL impairment.

**Figure 2:**
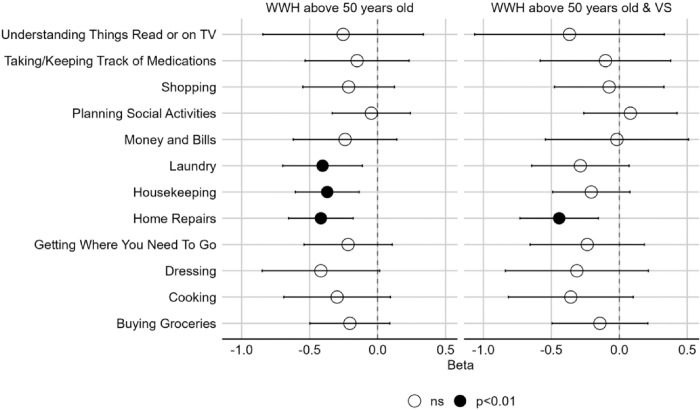
Associations between motor function and instrumental activities of daily living (IADL) in older [>50 years of age] women with HIV (WWH) and virally-suppressed (VS) women with HIV (WWH). A negative beta coefficient from the models indicate that better cognition is associated with a decrease in IADL impairment.

**Figure 3:**
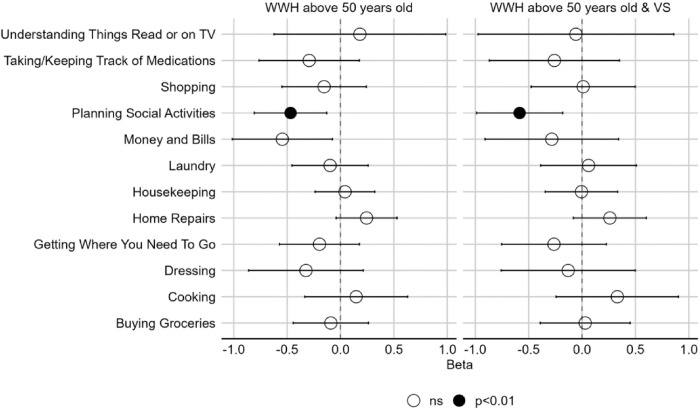
Associations between executive function and instrumental activities of daily living (IADL) in older [>50 years of age] women with HIV (WWH) and virally-suppressed (VS) women with HIV (WWH). A negative beta coefficient from the models indicate that better cognition is associated with a decrease in IADL impairment.

Among all participants, there were two domains relating to IADLs—motor function and executive function (Figures 1, 2, & 3). As the beta coefficients were negative, our models suggest that better motor function and executive function decrease the odds of impairment in IADLs. Alternatively, we can also state that poorer motor function and executive function increase the odds of impairment in IADLs. We used the initial interpretation throughout. In the older WWH overall, better motor function was associated with improved ability to conduct *home repairs* (OR=0.66, *p*=0.001), *housekeeping* (OR=0.69, *p*=0.002), and *laundry* (OR=0.67, *p*=0.007). Additionally, better executive function was associated with improved ability to *planning social activities* (OR=0.63, *p*=0.007)(Figure 2). Additionally, better executive function was associated with improved ability to *planning social activities* (OR=0.63, *p*=0.007)(Figure 3). Examining these associations among older virally suppressed-WWH, better motor function was associated with lower odds of impairment in *home repairs* (OR=0.64, *p*=0.003)(Figure 2) and better executive function was associated with lower odds of impairment in *planning social activities* (OR=0.56, *p*=0.005)(Figure 3).

## Discussion

Over the past 35 years, only ~ 1% of neuropsychological studies have focused on the relationship between cognition and everyday functioning [22]. Our study provides a unique overview of these relationships for older WWH and virally suppressed WWH. In this study, among the entire sample, there was a higher frequency of impaired everyday function in *working* (46%), *housekeeping* (15%), *home repairs* (12%), *planning social activities* (10%), and *laundry* (10%). This pattern is similar to that observed in another study of 277 PWH with HIV-Associated Neurocognitive Disorder, with the most frequently endorsed areas of impairment in *employment*, *planning and initiating social activities*, *housekeeping*, and *understanding TV programs and reading material* [23]. In a sample of 1,015 PWH from the AIDS Clinical Trials Group A5322 HAILO study (median age = 51 years) that also used the Lawton & Brody IADL scale, the most common impairments were *housekeeping* (48%), *transportation* (“getting to where you need to go”; 36%), and *shopping* (28%) [11]. Our WIHS sample did not have the same degree of impairments. Yet, these studies do show that subtle cognitive functioning is associated with impairments in everyday functioning.

We also examined which cognitive domain was most predictive of impairments in everyday functioning. Poorer motor functioning consistently predicted poorer function in *home repairs*, *housekeeping*, and *laundry*. All of these everyday activities require a certain level of dexterity and maneuverability of objects (i.e., folding a shirt, washing a dish, working with tools), such that poor motor function, as assessed by the groove pegboard task, could serve as a predictor of performing these everyday tasks. As PWH age, they will be more at risk of developing extrapyramidal motor signs than their HIV-negative counterparts. In fact, it has been shown that such mild extrapyramidal motor signs can contribute to poorer activities of daily living in older adults with HIV [24]. In addition, peripheral neuropathy (31.3%) [25] and arthritis (i.e., radiographic hand osteoarthritis, 55.8%) [26] are common in HIV, which can also affect neuropsychological test performance and everyday functioning [27]. Such factors may account for this relationship observed between motor functioning and impairments in these specific everyday activities.

The other major finding was that poorer executive function predicted poorer *planning social activities*. Several studies have noted a higher frequency of limitations in planning and initiating social activities on self-rated measures of daily living among functionally impaired PWH [23]. Difficulties with *planning social activities* were also the highest self-reported impairment in 138 functionally impaired PWH [28]. Fazeli et al. examined the role of episodic memory in functional dependence of younger and older PWH [13] and found among the younger PWH group (< 50 years) classified as functionally dependent, 68% reported an impairment in *social activities* as did 65% of the functionally dependent older PWH group. Analysis of performance on two tests of memory (CVLT and WMS Logical memory subtest) revealed that IADL dependence was associated with shallow encoding and forgetting. An impairment in *planning social activities* has implications for PWH as they age. Social relationships are crucial to healthy aging and are associated with lower mortality, better health, and higher self-reported well-being and quality of life, while social isolation has been associated with cognitive decline [29]. These findings had implications during the COVID-19 pandemic. Such social isolation during the pandemic coupled with the lack of social resources and HIV stigma could have had significantly impacted the ability to plan social events, leading to social isolation and further cognitive decline [30].

### Strengths and Limitations

Several methodological strengths are noted in this study. First, this is a large sample of older WWH who represent sites across the United States allowing these results to be generalizable to other older WWH. Second, the neuropsychological battery used has been validated and is established. Third, this study is one of the first to look at the Lawton & Brody IADL scale in an item-by-item analysis which represents a novel contribution to the literature; clearly different everyday functions require a different combination of cognitive abilities (e.g., driving performance [12]). Finally, the Lawton & Brody IADL scale self-assessment of everyday functioning is well accepted in the literature [31]. Overall, the innovation of this study is that it is the first HIV-related IADL article generated by the Women’s Interagency HIV Study to compare a large number of older WWH and virally suppressed WWH on cognitive differences in self-rated performance in specific IADLs; a further innovation of this includes: 1) controlling for a large number of covariates related to cognition and IADLs, 2) conducting an analysis in those virally suppressed; and 3) examining those over 50 years old.

Despite study strengths, the primary limitation is that the Lawton & Brody IADL scale is subject to recall bias and is not designed to be an objective performance-based measures of everyday functioning. Studies have suggested that adults may overestimate or underestimate their IADL ability compared to their actual performance [32]. For example, in a sample of 236 PWH 40 + years old, Jacob et al. [33] administered both a subjective (self-report) measure of IADLs (i.e., Lawton & Brody) and an objective laboratory-based performance measure of everyday functioning (i.e., Timed Instrumental Activities of Daily Living Test). From these measures, researchers calculated subjective/objective discrepancy scores. Nearly 58% demonstrated a discrepancy between subjective and objective performance, with many either over- or under-estimating their ability to performance IADL tasks. Inaccurate self-reporters were more likely to have poorer cognitive ability.

Another limitation of the Lawton & Brody IADL scale is that it does not capture the use of internet-based household IADLs such as internet shopping, handling money and bills, planning social activities, and other activities that are frequently handled virtually. As internet-based household IADLs become more integral in everyday functioning, the complexity of such computerized IADLs might be compromised by those with cognitive impairment. Woods et al. examined performance on internet-based household IADLs such as internet shopping and banking in 93 PWH (43 with HIV-Associated Neurocognitive Disorder) and 42 neurocognitively normal people without HIV [34]. These researchers found that those with HIV-Associated Neurocognitive Disorder experienced lower internet-based task scores and that such scores correlated with poorer motor skills, executive function, numeracy, and episodic memory.

Another limitation is that sex differences were not examined, as the data for men were not available. Clearly, sex differences could exist and influence the relationships between cognition and IADLs, especially when considering traditional sex differences in some ADLs such as *shopping, housekeeping*, and *childcare* which may bias one’s exposure and perception of engaging in these activities. In fact, this Lawton & Brody IADL measure was modified to include *childcare* as this study targeted women.

Finally, we cast a wide net in exploring associations between individual items of the Lawton & Brody IADL with the seven cognitive domains in different subgroups, thus creating multiple comparisons thus inflating type 1 error rate. Unfortunately, using strict alpha correction (i.e., Bonferroni) will also artificially exclude actual relationships (i.e., inflating type 2 error rate). From a practical and exploratory perspective, that is why significance was set at *P* ≤ 0.01, so that we could identify associations of interest while balancing both type 1/type 2 error rate.

### Future Directions

Building on these results, there are several research vectors. First, the data reported in this article were weighted longitudinal data; however, the WIHS cohort is a rich and complex longitudinal data set. These data can be further examined in other ways such as examining distinct cognitive profiles. Dastgheyb et al. identified five distinct cognitive impairment profiles in WWH in the WIHS which are: 1) speed profile; 2) sequencing (executive function) profile, 3) learning + memory profile; 4) learning + recognition profile; and 5) executive function + learning + attention + processing speed profile. Perhaps those with different cognitive impairment profiles also have different patterns of IADL impairment over time [35].

Second, cognitive training may be used to target the cognitive domains associated with impairments in everyday functioning. In a systematic review of 13 cognitive training studies in PWH, researchers concluded that, in general, cognitive training improved performance in the targeted cognitive domain that was targeted (i.e., executive functioning training improved executive functioning ability) [36]. In some studies, cognitive training that targets improvement on one cognitive domain also transferred to improvement in measures of everyday functioning. For example, in a randomized sample of 46 PWH (no-contact control group or speed of processing (SOP) training group), those in the SOP training group demonstrated improved performance on the Timed Instrumental Activities of Daily Living (TIADL) test [37]. Perhaps individualized cognitive domain training that targets motor function may improve housekeeping and other such IADLs; likewise, executive functioning training may improve the ability to plan social activities [38].

## Conclusion

As PWH age, concerns mount that cognitive decline will contribute to declines in everyday functioning as well. These declines increase the burden on social support systems and can have wide reaching public health impact. In fact, as WWH age, many of them will be caregivers to family and friends as well, further contributing to poorer later life outcomes and quality of life [39].

## Figures and Tables

**Table 1.** Participant Characteristics in WWH and Virallly-Suppressed WWH

Variables	All WWH Age >= 50 *n* (%)	VS WWH Age >= 50 *n* (%)	*p*-value*
***Sample size***	754	637	*na*
***Number of contributing visits to the analysis***	1,457	1,080	
***Enrollment Wave***			0.92
1994–1995	357 (47)	293 (46)	
2001–2002	127 (17)	113 (18)	
2011–2012	76 (10)	61 (10)	
2013–2015	194 (26)	170 (27)	
***Clinic site locations***			
Chicago, DC, LA, NY, SF	558 (74)	466 (73)	0.76
Atlanta, Birmingham, Chapel Hill, Jackson	196 (26)	171 (27)	0.76
***Sociodemographic***			
Age, *M (SD)*	55.03 (4.96)	55.23 (5.00)	0.46
Years of Education, *M (SDD*	12.49 (2.96)	12.45 (3.01)	0.79
Race			0.99
Black non-Hispanic	533 (71)	447 (70)	
Hispanic	88 (12)	77 (12)	
Other	23 (3)	21 (3)	
White	110 (15)	92 (14)	
Annual income < $12,000 per year	405 (54)	327 (51)	0.39
Employed	234 (31)	207 (32)	0.56
Insured	750 (99)	633 (99)	1.00
***Mental health and substance use***			
Depressive symptoms	11.56 (11.13)	11.25 (11.03)	0.59
Recent			
Crack	45 (6)	30 (5)	0.34
Cocaine	20 (3)	15 (2)	0.86
Heroin	11 (1)	4 (1)	0.19
Marijuana	140 (19)	111 (17)	0.62
Current Smoker	300 (40)	240 (38)	0.44
***HIV-related clinical characteristics***			
Nadir CD4	277.9 (200.42)	281.94 (195.44)	0.70
Current CD4	665.98 (338.55)	695.07 (315.69)	0.10
Years of effective ART	12.9 (6.67)	13.08 (6.75)	0.63
Viral Load (log)	3.6 (1.61)	-	
ART adherence (≥ than 95%)	653 (87)	574 (90)	0.05*
Common ART drugs Emtricitabine (FTC)	519 (69)	442 (69)	0.86
Tenofovir (TDF)	387 (51)	317 (50)	0.59
Ritonavir (RTV)	250 (33)	191 (30)	0.22

*Note.* T test is used for comparison of onctinuous variables. Fisher’s exact test is used for categorical variables.

* *p*-value < 0.05.

**Table 2.** Summary of Lawton & Brody IADL Items and Cognitive Function Values

Variables	All WWH Age >= 50 *n* (%)	VS WWH Age >= 50 *n* (%)	*P*-value*
***lADLs***			
Total Score	550 (38)	375 (35)	0.12
Housekeeping	212 (15)	129 (12)	0.06
Money and bills	56 (4)	32 (3)	0.27
Buying groceries	128 (9)	79 (7)	0.19
Cooking	62 (4)	36 (3)	0.25
Planning social activities	145 (10)	93 (9)	0.27
Understanding things read or watch on TV	20 (1)	13 (1)	0.86
Getting where you need to go	81 (6)	47 (4)	0.20
Home repairs	181 (12)	124 (11)	0.50
Dressing	39 (3)	25 (2)	0.61
Shopping	94 (6)	57 (5)	0.24
Laundry	144 (10)	77 (7)	0.02*
Taking/keeping track of medications	52 (4)	34 (3)	0.58
Using the phone	2 (<1)	1(<1)	1.00
Bathing	12 (1)	4 (<1)	0.21
Taking care of children/grandchildren	31 (2)	13 (1)	0.09
Working	674 (46)	467 (43)	0.14

***Cognitive function***	*M (SD)*	*M (SD)*	
Verbal learning	49.42 (10.35)	50.05 (10.17)	0.12
Verbal memory	49.49 (10.34)	49.75 (10.36)	0.52
Verbal fluency	49.34 (9.52)	49.70 (9.63)	0.35
Attention/Working memory	48.37 (9.67)	48.43 (9.59)	0.87
Psychomotor speed	49.40 (9.48)	49.69 (9.47)	0.44
Executive function	48.87 (9.99)	49.34 (9.87)	0.24
Motor function	49.93 (10.53)	50.28 (10.26)	0.40

*Note.* T test is used for comparison of onctinuous variables. Fisher’s exact test is used for categorical variables.

* *p*-value < 0.05.

## Data Availability

All data are publically available. Data analysis of data are available upon request.
